# Comparative genomics analysis of endangered wild Egyptian *Moringa peregrina* (Forssk.) Fiori plastome, with implications for the evolution of Brassicales order

**DOI:** 10.3389/fgene.2023.1131644

**Published:** 2023-03-13

**Authors:** Heba A. M. AbdAlla, Vincent Okelo Wanga, Elijah Mbandi Mkala, Sara Getachew Amenu, Mohamed Hamdy Amar, Lingyun Chen, Qing-Feng Wang

**Affiliations:** ^1^ CAS Key Laboratory of Plant Germplasm Enhancement and Specialty Agriculture, Wuhan Botanical Garden, Chinese Academy of Sciences, Wuhan, China; ^2^ Plant Biodiversity and Evolution Research Group, University of Chinese Academy of Sciences, Beijing, China; ^3^ Botany Department, Agriculture and Biological Institute, National Research Centre, Giza, Egypt; ^4^ Sino-Africa Joint Research Center, Chinese Academy of Sciences, Wuhan, China; ^5^ Egyptian Deserts Gene Bank, Desert Research Center, Cairo, Egypt; ^6^ Department of Resources Science of Traditional Chinese Medicines, School of Traditional Chinese Pharmacy, China Pharmaceutical University, Nanjing, China

**Keywords:** comparative genomics, endangered, wild egyptian moringa peregrina, plastome, phylogeny, estimation time

## Abstract

*Moringa* is a mono-genus belonging to the Moringaceae family, which includes 13 species. Among them, *Moringa peregrina* is plant species native to the Arabian Peninsula, Southern Sinai in Egypt, and the Horn of Africa, and comprehensive studies on its nutritional, industrial, and medicinal values have been performed. Herein, we sequenced and analyzed the initial complete chloroplast genome of *Moringa peregrina*. Concurrently, we analyzed the new chloroplast genome along with 25 chloroplast genomes related to species representing eight families in the Brassicales order. The results indicate that the plastome sequence of *M. peregrina* consists of 131 genes, with an average GC content of 39.23%. There is a disparity in the IR regions of the 26 species ranging from 25,804 to 31,477 bp. Plastome structural variations generated 20 hotspot regions that could be considered prospective DNA barcode locations in the Brassicales order. Tandem repeats and SSR structures are reported as significant evidence of structural variations among the 26 tested specimens. Furthermore, selective pressure analysis was performed to estimate the substitution rate within the Moringaceae family, which revealing that the *ndhA* and *accD* genes are under positive selective pressure. The phylogenetic analysis of the Brassicales order produced an accurate monophyletic annotation cluster of the Moringaceae and Capparaceae species, offering unambiguous identification without overlapping groups between *M. oleifera* and *M. peregrina*, which are genetically strongly associated. Divergence time estimation suggests that the two *Moringa* species recently diversified, 0.467 Ma. Our findings highlight the first complete plastome of the Egyptian wild-type of *M. peregrina*, which can be used for determining plastome phylogenetic relationships and systematic evolution history within studies on the Moringaceae family.

## Introduction

The Moringaceae is dicotyledonous flowering plants family consisting of 13 species (Olson, 2002). The family is commonly known as the horseradish or drumstick family (Abd Rani et al., 2018). It is classified under the morphologically diverse order Brassicales together with Brassicaceae, Salvadoraceae, Capparaceae, Cleomaceae, Pentadiplandraceae, Akaniaceceae, and Caricaceae (Group et al., 2016; Leite and Castilho, 2017). Species of the Moringaceae family have a wide distribution mostly in the tropical and sub-tropical regions occurring mainly in India, China, mainland Africa and the Arabian Peninsula (Fahey, 2005; Amaglo et al., 2010; Moerman, 2013; Abd Rani et al., 2018). *Moringa* species are primarily shrubs or subshrubs with opposite leaves, tuberous roots, and zygomorphic flowers (Olson and Committee, 1993). *Moringa oleifera* species are recorded as native to India, Pakistan, and Nepal (Padayachee and Baijnath, 2012; Liu et al., 2019), whereas *M. peregrina* (Habb El Yasar) is reported to be an Egyptian native tree with a wide biogeographic distribution along the Red Sea coast up to Sinai Mountains in the Arabian desert (Boopathi and Abubakar, 2021). Recently, the *Moringa* genus gained attention due to the rich pharmaceutical traits used against more than 300 health disorders (Ebert et al., 2019). In previous studies, *M. oleifera* has primarily been used in terms of phytochemistry (El Sohaimy et al., 2015; Jaja-Chimedza et al., 2017), and some molecular studies were solved *M*. *oleifera* using genetic markers (Barbosa et al., 2019; Sodvadiya et al., 2020). The unique seed oil composition (Ben oil) extracted from *M. peregrina* has gained scientific interest (Al-kahtani, 1995).

Egyptian *M. oleifera* and *M. peregrina* extracts were reported to have anti-cancer and anti-microbial phytochemicals according to GC-MS technology results (Mansour et al., 2019). In addition, its essential oils were reported to have rich anti-cancer components in a study on the treatment of zebrafish embryos with their seed extracts (Elsayed et al., 2020). Additionally, bark extract was estimated to enhance the ratio of B6 and cytochrome genes in under-experienced rats (Rizk et al., 2021). Recently, Menon et al., 2021, reported that *M. peregrina* leaf extract is rich in anti-inflammatory and antioxidant components, which can improve prostatic hyperplasia in rat glands.

Previous studies on*Moringa* species have used two morphological parameters: The flower’s irregular and winged seeds (Verdcourt, 1986) and habitats (Olson and Carlquist, 2001). Previous taxonomic studies classified Moringaceae and Caricaceae families into the subclade for the higher order Capparales (Karol et al., 1999). Recently, both families were classified as families belonging to the order of Brassicales, counted as highly diversified and advanced among angiosperms, comprising 17 families (Group, 2009). However, *M. oleifera* and *M. peregrina* are phylogenetically closely related (Verdcourt, 1986; Olson and Carlquist, 2001). However, *M. peregrina* has significant medicinal value.

In a molecular phylogenetic study, *M. oleifera* and *M. peregrina* showed a close relationship based on the genes of seven *Moringa* species (Abdel-Hameed, 2015). Additionally, phylogenetic analyses using *rbcL* showed a close relationship between the sister families of the Moringaceae and the Caricaceae (Karol et al., 1999). However, a limited number of studies have focused on systematic analyses in Brassicales. Next-generation sequencing technology (NGS) has made available various genomic resources during the past 2 decades, including the complete cpDNA sequence. The plastome has lower substitution rates than the mitochondrial and nuclear genomes used in several systematic plant studies (Zhang et al., 2012; Asaf et al., 2017). In addition, plastome sequences have become a cheap and practical resource for non-model genotypes and for predicting complete phylogenetic attitude and gene evolution (Asaf et al., 2016; Khan et al., 2019). At present, about 6,500 plastomes of pharmaceutically and commercially important species are currently available in the chloroplast genome database (CPGDB) (Singh et al., 2020). Among them, a limited number of Brassicales order plastomes had available sequences in the National Center for Biotechnology Information (NCBI) databases. However, the Moringaceae are a complex family with uncertainties in classical taxonomy due to floral and morphological similarities (Olson and Carlquist, 2001). Regardless of the undisputed uniqueness of the family, the phylogenetic dataset is still a rarity in its species. The only reported plastome in the National Center for Biotechnology Information (NCBI) database among the 13 species of the Moringaceae family is that of *M. oleifera* (Lin et al., 2019; Yang et al., 2019). To date, there is no study has reported a complete plastome genome sequence for an Egyptian wild *Moringa* species. Therefore, further comparative research on the Moringaceae family still needs to be conducted.

Here, we report a newly, firstly completed plastome of the endangered wild type of Egyptian *Moringa peregrina* to improve our understanding of plastome characteristics, structural diversity, and evolution within the Brassicales order. The main objectives of this study were to 1) assemble and annotate the genome structures of *Moringa peregrina;* 2) reveal structural and size variations and trace the evolutionary patterns of the IR boundaries in the plastomes of *M. peregrina* and 25 species belonging to eight families of the Brassicales order, as placeholders 3) recognize highly variable hotspots of the plastome for the advanced phylogenetic evolutionary and systematic revision of the Moringaceae placeholders; and 4) assess the phylogenetic framework and finally infer the divergence time estimates and adaptive evolution of species in this section, which has yet to be determined. We selected 24 species due to the limited Moringaceae species studied so far, as *M. oleifera* has gained all research attention so far (Lin et al., 2019; Liu et al., 2019). Moreover, we aimed to increase the number of species in our analysis with respect to the previous paper, where only 10 species of the Brassicales order were studied (Khan et al., 2021). Therefore, the present genomic information’ provides vital genetic datasets to determine phylogenetic relationships, and genome diversity in future genetic evolution-related studies of *M. peregrina* in complex Brassicales order families as a part of angiosperm plants.

## Materials and methods

### DNA extraction and sequencing

Fresh young leaves of the wild type of *M. peregrina* were collected from the natural habitat (Saint Catherine, Egypt (Wadi Zaghra) (28°39′05″N 34°189.7″E) in collaboration with seed collection team of Desert research center, Cairo, Egypt. Leaves were dried using silica gel (Wilkie et al., 2013). About 0.5 g of dried leaves was used for DNA extraction using a modified CTAB protocol (Doyle, 1991). Sequencing was performed by Novogene company (Beijing, China) using the Illumina paired-end technology platform.

### Data processes, assembly, annotation, and plastome structural features

After removing low-quality reads and adapters, high-quality reads were assembled using the GetOrgenelle ver. 1.7.4 application (Jin et al., 2020), followed by manual correction using Sequin Ver.16.0 (Liu et al., 2012). Plastid Genome Annotator (PGA) software was used for gene annotation (Qu et al., 2019) with *M. oleifera* as the reference (NC_041432). Geneious prime ver.8.0.4 (Kearse et al., 2012) and GeSeq online tool (https://chlorobox.mpimp-golm.mpg.de/geseq.html) (Tillich et al., 2017) were used to manually check and edit the genome and submit it to the NCBI database under gene bank accession number ON855355. The circular plastome was drawn using Organelle Genome DRAW (OGDRAW) online software (https://chlorobox.mpimp-golm.mpg.de) (Greiner et al., 2019). The comparative genomes were obtained for *M. peregrina* and 25 related species in shuffle-LAGAN mode (Brudno et al., 2003). MVISITA software (https://genome.lbl.gov) (Frazer et al., 2004) used *Arabidopsis thaliana (*NC_000932) as the annotation reference.

### Repeats and codon bias analyses

For repeat analysis and codon bias analysis, the REPuter online application (https://bibiserv.cebitec.uni-bielefeld.de/reputer/) was used to identify forward, reverse, palindromic, and complement repeat types (Kurtz et al., 2001). Specifically, the framework for the smallest repeats size was 30 bp, and the structure reported more than 90% (hamming distance of 3). Single sequence repeat (SSRs) motifs were determined using the revised online software MISA (http://misaweb.ipk-gatersleben.de/) (Beier et al., 2017). Parameters for SSR motifs were 10, 5, 4, 3, 3, and three for mono-, di-, tri-, tetra-, penta-, and hexanucleotide types, respectively. The RSCU program merged in Phylosuite ver.1.2.2 (Zhang et al., 2020) was used for drawing the codon bias ratio. Junction sites among plastomes were visualized using the IRscope online application (https://irscope.shinyapps.io/irapp/) (Amiryousefi et al., 2018).

### Selective pressure analysis

The substitution rate within *Moringa* species was estimated using 74 protein-coding genes from the cp plastomes of two *Moringa* representative species (*M. peregrina* and *M. oleifera*). The regions were extracted using Phylosuite ver. 1.2.2 (Zhang et al., 2020). Stop codons were manually cut and removed, and the gap within the sequences was removed using Gblocks implanted in Phylosuite. MAFFT ver.7, included in Phylosuite ver. 1.2.2, was used to align the combined files after stop codons and gaps were removed. We manually converted the aligned files by saving them in AXT format. The non-synonymous (Ka), and synonymous (Ks) rates of the 74 protein-coding genes, as well as the Ka/Ks ratio of each region, were calculated using Ka/Ks calculator ver. 2.0, and maximum likelihood methods Ma and Ms were selected (Li et al., 2009).

### Phylogenetic analysis

The present study used 34 plastomes belonging to the Brassicales order for plastome phylogenetic relationship determination. According to a previous study, the two *Gossypium* species were used as out-groups (Khan et al., 2021). Further, 54 protein-coding sequences (CDSs) were aligned in groups with MAFFT ver. 7.313 (Supplementary Table S1) (Katoh and Standley, 2013). MAFFT was applied to compare gene arrangement and reveal missing gene alignment after multiple structure annotation and was utilized to estimate pairwise-sequence variations. Furthermore, to evaluate phylogenies across species, we employed the maximum likelihood (ML) and Bayesian inference (BI) models described using IQ-tree ver.1.6.8 and Bayesian (MrBayes ver.3.2.6) merged into Phylosuite ver.1.2.2 (Zhang et al., 2020). Model Finder was used to estimate the fittest model using the Bayesian information criterion (BIC) (Kalyaanamoorthy et al., 2017). The fittest model for ML analysis was GTR + R3+F, while that for Bayesian analysis was JTT + F + R3. Moreover, the JTT + F + R3 models and GTR + R3+F were run for 1,000 rapid bootstrapping replicates using ultrafast bootstraps (Minh et al., 2013) and the Shimodaira–Hasegawa like approximate likelihood-ratio test (Guindon et al., 2010). Finally, Figtree ver.1.4.2 (Drummond et al., 2012) was used to visualize the phylogenetic topology results.

### Divergence time estimation

Divergence date estimates were evaluated using Bayesian methods under a relaxed molecular clock to account for rate variation among lineages (Drummond et al., 2012). In BEAST ver. 1.8.0, we used an uncorrelated relaxed lognormal model of rate evolution to simultaneously estimate phylogeny and divergence times. To account for the reasonable assumption that the calibrated node 0) fossil was not older than the fossil’s first recorded age, fossil calibrations were constrained as follows: Normal priors, mean, 125; std 1.0; and the 95% upper limit equal to the stratigraphic age plus 10%. (Ho and Phillips, 2009). To constrain the age of Brassicales, the second node was calibrated using a secondary calibration (lognormal priors; offset 114; Mean 0.5; std 1.0 (Cardinal-McTeague et al., 2016). To obtain the exact diversification period of *Moringa peregrina* and *Moringa oleifera*, we used BEAUti ver. 1.8.0 to obtain a partitioned 54-gene data set to generate XML files. The rate of molecular evolution and rate variation parameters were estimated using an uncorrelated relaxed clock model. The tree model was tested using the Yule process of speciation (Yule, 1925), which began with a randomly generated tree. Brassicales were given uniform height priors ranging from 0 to 125 Mya, implying that those nodes could not be older than the earliest recorded evidence of eudicot fossils (Brenner, 1996; Sun et al., 2011). The node’ prior time constraints were chosen using the lognormal distribution of mean and standard deviation set at the mean and median limits, and the GTR +1 + G substitution model was set as the nucleotide substitution model. BEAST2 in XSEDE ver. 1.8.0 on the CIPRES web server was used to estimate the dating time. The MCMC analysis was run for 10 million generations with sampling every 1,000 generations. Further, Tracer ver. 1.7.1 was used to evaluate the runs for convergence and ESS. If convergence and ESS (>200) were met, runs were combined using Log Combiner ver.1.8.0 after a 25% (10 million generations) burn-in and summarized with Tree Annotator ver. 1.8.0 to produce a maximum clade credibility tree with height median ages. The tree result file was visualized with Figtree ver. 1.4.4 to show mean divergence time approximations with 95% HPD intervals.

## Results

### Comparative analyses of *Moringa peregrina* plastome structural features

**FIGURE 1:**
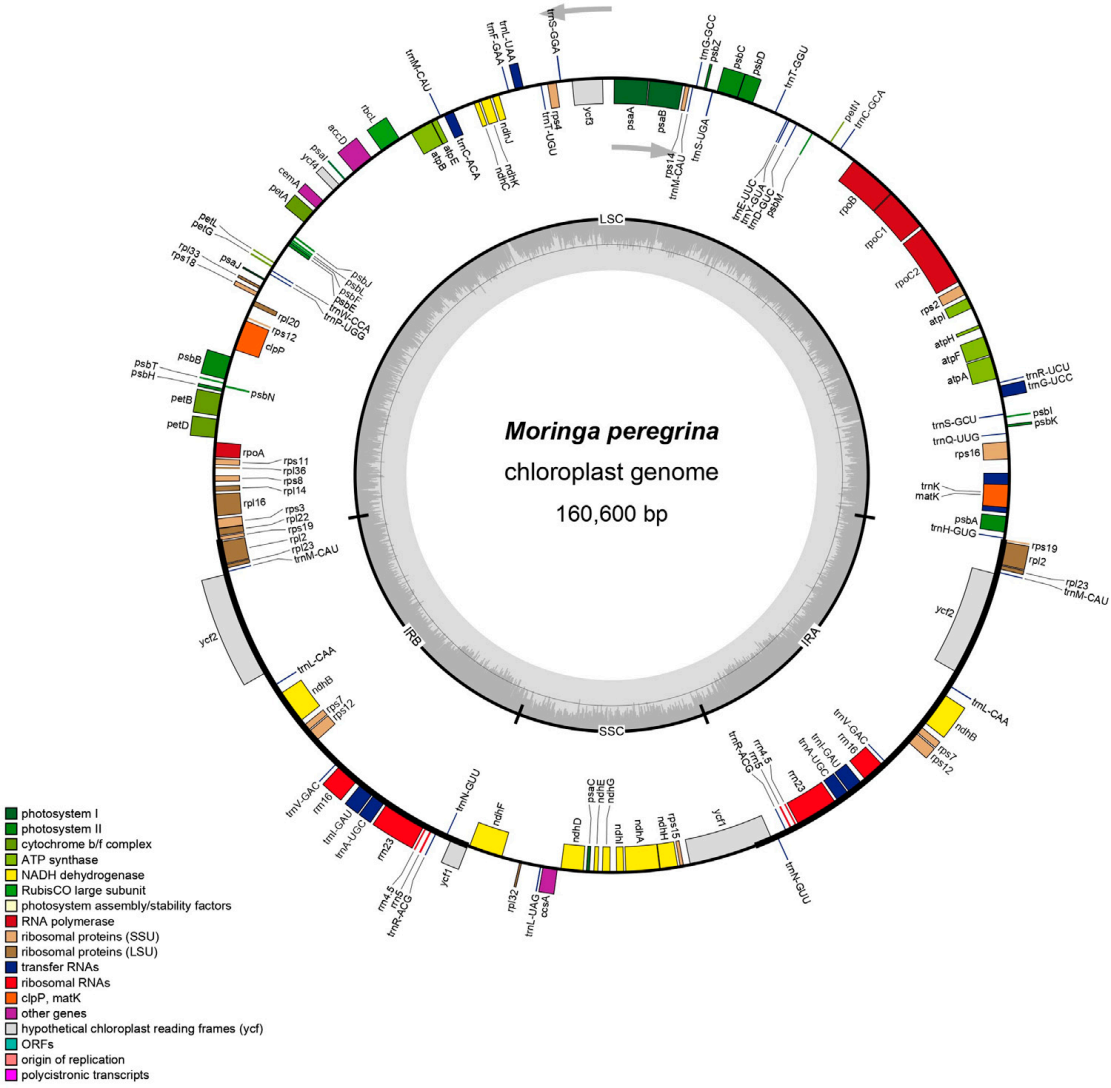
The map of *M. peregrina* plastome. The genes are classifield into the circle in the clockwise direction for inside genes and the anticlockwise direction for outside genes. Every color belongs to the same gene function group. IRa & IRb extant shown in the thick lines, which divided plastome sequence with LSC and SSC.

The plastome assembly of *M. peregrina* exhibits a double-stranded circular DNA molecule of 160,600 bp in length. It has a quadripartite natural structure that comprises LSC (88,577 bp) and SSC region (18,883 bp) divided by two inverted repeats (26,250 bp) (Figure 1; Table 1). The plastome sequence of *M. peregrina* consists of 131 genes, comprising 87 protein-coding genes, four rRNA genes; and 30 genes, encoding tRNA, that are duplicated (Table 1; Figure 1); (9 large and 12 small ribosomal subunits; four DNA-dependent RNA polymerases; and 10 genes decoding other proteins (Table 2). In detail, 18 genes containing determined introns in *M. peregrina* plastome comprise seven tRNA genes and 11 protein-coding genes, whereas the *ycf3* and *clpP* genes have two introns (Table 2). The largest intergenic exon region belongs to the *tRNA-UUU* gene (2,541 bp), while the smallest exon region is that of *trnL-UAA* gene (Table 2). When comparing the 26 related plastomes, four common regions include a small single-copy (SSC) part, large single copy (LSC) part, and copy inverted repeat (IR) parts (Table 3). The plastome structure length ranges from 152,860 bp (Brassica napus) to 160,600 bp (*M. oleifera* and *M. peregrina*). All related plastomes show a typical quadripartite structure, consisting of a couple of IR regions (25,804–31,477 bp) divided by the LSC region (83,030–88,749 bp) and the SSC region (9.631–18,883 bp) (Table 3). Moreover, the total number of genes ranges from 127 to 132, and protein-coding genes range from 73 to 87. In addition, tRNA genes range from 35 to 38, and rRNA genes contain eight genes; and only *A. tetracantha*has 7 (Table 3).

**TABLE 1:** Genes composition in *Moringa peregrina* plastome.

Gene’s function	Genes classified	Genes name	Numbers
Photosynthesis genes	ATP synthase subunits	*atpA, atpB, atpE, atpF, atpH, atpI*	6
	Phyotosystem II subunits	*psbA, psbB, psbC, psbD, psbE, psbF, psbI, psbJ, psbK, psbL, psbH, psbM, psbN, psbT, psbZ, ycf3*	16
	NADH-dehydrogenase subunits	ndhA, ndhB(*2), ndhC, ndhD, ndhE, ndhF, ndhG, ndhH, ndhI, ndhJ, ndhK	12
	Cytochrome b/f complex subunits	*petA, petB, petD, petG, petL, petN*	6
	Phytosystem I subunits	*psaA, psaB, psaC, psaI, psaJ*	5
	Rubisco (rbcL) subunits	*rbcL*	1
Self-replication	Large ribosome subunits	rpl14, rpl16, rpl2(*2), rpl20(*2), rpl22, rpl23(*2), rpl32, rpl33, rpl36	12
	DNA dependent RNA polymerase subunits	*rpoA, rpoB, rpCo1, rpoC2*	4
	Small ribosome subunits	rps11, rps12(*2), rps14, rps15, rps16, rps18, rps19, rps2, rps3, rps4, rps7(*2), rps8	14
	tRNA genes	tRNAI-CAU (*2), tRNAL-CAA, tRNAV-GAC(*2)	36
		tRNAI-GAU (*2), tRNAA-UGC(*2), tRNAP-UGG	
		*tRNAW-CCA, tRNAM-CAU, tRNAV-UAC, tRNAF-GAA*	
		*tRNAL-UAA, trnT-UGU, trnS-GGA, trnfM-CAU*	
		*tRNAG-GCC, tRNAS-UGA, tRNAT-GGU, tRNAE-UUC*	
		*tRNAY-GUA, tRNAD-GUC, tRNAC-GCA, tRNAR-UCU*	
		*tRNAG-UCC, tRNAS-GCU, tRNAQ-UUG, tRNAK-UUU*	
		tRNAH-GUG, tRNAR-ACG (*2), tRNAN-GUU(*2)	
		*tRNAL-UAG*	
	rRNA genes	rRNA 4.5 (*2), rRNA 5(*2), rRNA 16(*2), rRNA 23 (*2)	8
Other genes	Acetyl CoA- carboxylase subunit	*aacD*	1
	C- type cytochrome synthesis gene subunit	*ccsA*	1
	Envelop membrane protein subunit	*cemA*	1
	Protease subunit	*clpP*	1
	Maturase subunit	*matK*	1
Unknown conserved	Open reading frams subunits	ycf15(*2,) ycf2(*2), ycf4,ycf1	6
Total			131

Note: *, duplicated genes.

**TABLE 2:** The length of intragenic regions.

Gene	Strand	Start	End	ExonI	Exon II	Exon III	Intron II	Exon III
*trnK- UUU*	−	1,692	4,304	37	2,541	35		
*rps 16*	−	5,164	6,329	41	899	266		
*atp F*	−	12,000	13,290	160	721	410		
*ycf 3*	−	44,855	46,863	124	732	230	770	153
*trn L- UAA*	+	49,985	50,564	38	493	49		
*trn V - UAC*	−	54,752	55,431	39	604	37		
*clpP*	−	74,187	76,227	71	850	294	600	226
*pet B*	+	79,174	80,621	6	788	654		
*rpl 16*	−	85,626	87,094	9	1,061	399		
*rp12*	−	88,733	90,222	391	665	434		
*ndh B*	−	99,335	101,555	775	688	758		
*trnI-GAU*	+	107,161	108,172	42	936	34		
*trnA-UGC*	+	108,237	109,105	38	796	34		
*ndhA*	−	125,480	127,708	553	1,137	539		
*trnA-UGC*	−	140,073	140,941	38	796	35		
*trnI-GAU*	−	141,006	142,017	42	936	34		
*ndhB*	+	147,623	149,843	775	688	758		
*rp12*	+	158,956	160,445	391	665	434		

**TABLE 3:** Summary of annotated features of 26 relative’s plastome.

Species	Accession number	Family name	CP genome length (bp)	LSC length (bp)	SSC length (bp)	IR length(bp)	Genes number	Protein- coding genes number	tRNA numbers	rRNA numbers
*Aethionema arabicum*	NC_034367	Brassicaceae	154,234	83,401	17,716	26,558	128	83	37	8
*Aethionema grandiflorum*	NC_009266	Brassicaceae	154,243	83,473	17,748	26,511	129	84	37	8
*Akania lucens*	NC_049582	Akaniacaeae	158,188	86,743	18,631	26,407	132	86	37	8
*Arabidopsis halleri*	NC_034366	Brassicaceae	154,671	84,279	17,870	26,261	130	85	37	8
*Arabidopsis thaliana*	NC_000932	Brassicaceae	154,478	84,170	17,780	26,264	129	85	37	7
*Azima tetracantha*	NC_043808	Salvadoraceae	153,415	83,841	17,488	26,043	131	84	37	8
*Brassica napus*	NC_016734	Brassicaceae	152,860	83,030	17,762	26,035	132	87	37	8
*Brassica nigra*	NC_030450	Brassicaceae	153,633	83,552	17,695	26,193	132	87	37	8
*Brassica oleracea*	NC_041167	Brassicaceae	153,364	83,136	17,834	26,197	132	87	37	8
*Brassica rapa*	NC_040849	Brassicaceae	153,483	83,281	17,775	26,213	132	87	37	8
*Bretschneidera sinensis*	NC_037753	Akaniacaeae	159,004	86,910	18,820	26,637	132	87	37	8
*Capparis spinosa* var. *herbacea*	NC_047194	Capparaceae	159,216	86,631	9,631	31,477	127	73	37	8
*Capparis spinosa* var. *ovata*	NC_049616	Capparaceae	157,783	86,272	18,743	26,384	132	87	37	8
*Capparis spinosa* var. *spinosa*	NC_047193	Capparaceae	157,796	86,700	18,312	26,392	132	85	37	8
*Capsella grandiflora*	NC_028517	Brassicaceae	154,638	83,879	17,835	26,462	130	85	37	8
*Carica papaya*	NC_010323	Caricaceae	160,100	88,749	18,701	26,325	131	84	37	8
*Cleomella lutea*	NC_049613	Cleomaceae	154,124	83,700	18,114	26,155	132	84	37	8
*Crateva tapia*	NC_049621	Capparaceae	156,962	85,430	18,772	26,380	132	87	37	8
*Moringa oleifera*	NC_041432	Moringaceae	160,600	88.577	18,883	26,570	129	85	36	8
*Moringa peregrina*	ON855355	Moringaceae	160,600	88.577	18,883	26,570	131	87	36	8
*Pentadiplandra brazzeana*	NC_044667	Pentadiplandraceae	156,625	85,318	17,805	26,751	130	85	37	8
*Ricotia aucheri*	NC_039956	Brassicaceae	154,770	—	—	26,509	130	87	35	8
*Ricotia cretica*	NC_039958	Brassicaceae	154,188	83,274	18,169	26,372	132	85	37	8
*Salvadora persica*	NC_057955	Salvadoraceae	153,379	83,818	17,683	25,939	130	85	37	8
*Tarenaya hassleriana*	NC_034364	Cleomaceae	157,688	87,509	18,677	25,804	131	85	38	8
*Vasconcellea cundinamarcensis*	NC_049867	Caricaceae	158,712	87,991	17,841	26,440	130	84	37	8

### IR junction variations in *Moringa peregrina* plastome

**FIGURE 2:**
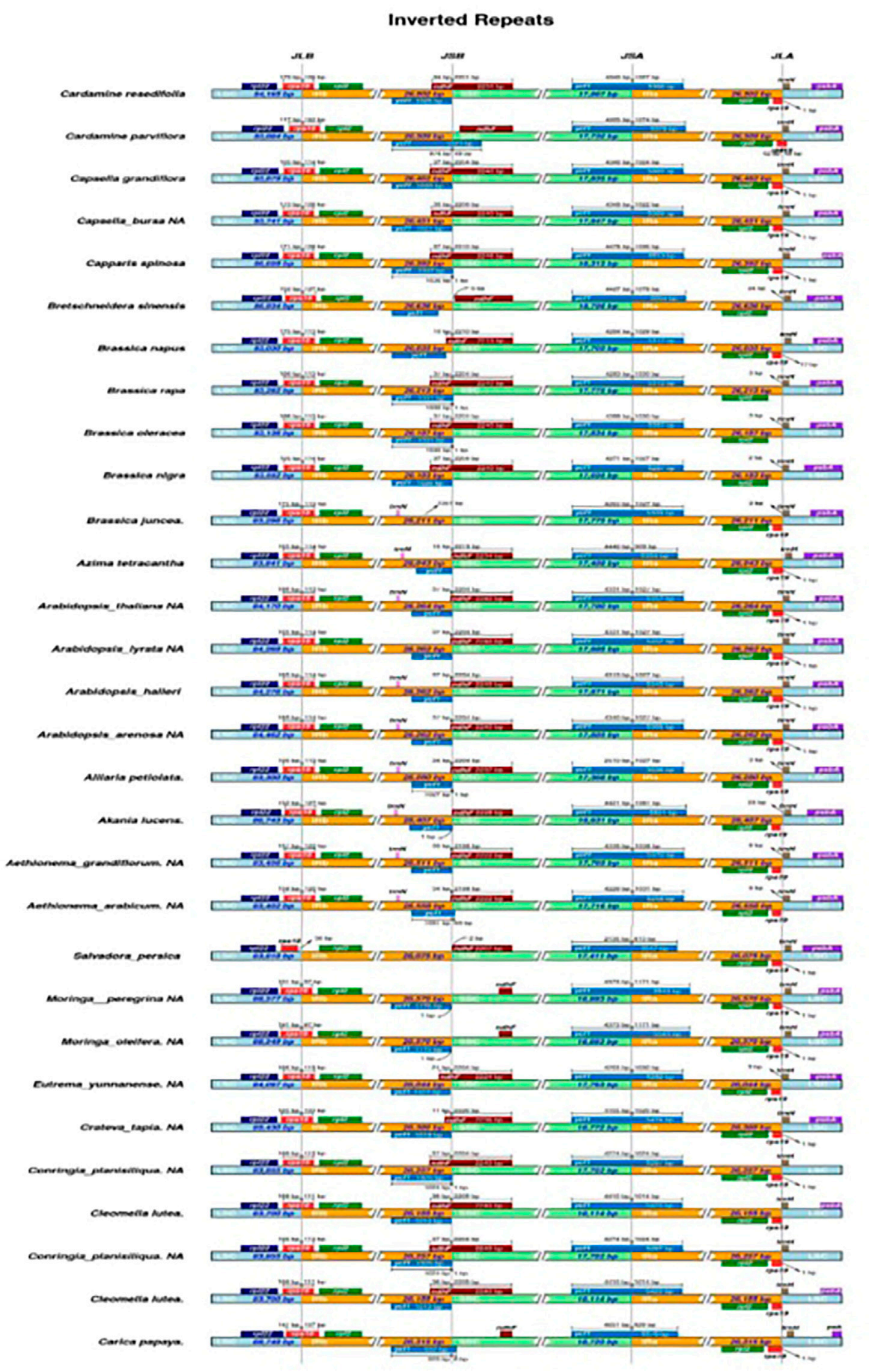
Distance between abjection genes and junctions of the small single copy(SSC), long single copy (LSS) and two inverted repearts regions (IR) of *M. peregrina* with 26 related species plastomic structures. The figure only shows the relatives changes at or near to IR/SC borders.

In most angiosperm plastome structures, the IR regions are the most conserved ones in whole-plastome structural regions. There is a positive correlation between IR length and plastome length. According to our study, the IR length of *M. peregrina* is similar to what previously reported angiosperm plastomes. The current study reported a close correlation between four junction regions (JLB, JSB, JSA, and JLA) and the three plastome regions (IR a, and b, LSS, and SSC) in *M. peregrina*. In addition, when considering the 25 tested specimens with related plastome sequences (Figure 2), the results indicate a disparity in the IR length regions of the 26 species, ranging from 25,804 bp (*Tarenaya hassleriana)* to 31,477 bp (*Capparis spinosa var herbacea)*. The results show that in *Salvadora persica*, the *rps19* gene was estimated to be 36 bp away from the JLB junction at the end of the LSC region; in contrast, in *Pentadiplandra brazzeana* the *rps19* gene was found to be in the IRa and IRb regions. In the *M. peregrina* plastome, the *ycf1* gene is at the JSA junction, with 4,373 bp in the SSC region and 1,171 bp in the IRa region, while *trnH* extends by 36 bp from the JLA junction toward the LSC region (Figure 2). Similarly, the *psbA* gene was found to be in the LSC region in the studied species. On the other hand, in *Pentadiplandra brazzeana,* the *rpl22* gene is in the IRb region. The position of the *rps19* gene in all related plastomes is close to that in *A. thaliana* except for the cases of *Salvadora persica* (where it can be found in the LSC region) and *Pentadiplandra brazzeana* (where it can be found in IRA and IRb regions). Regarding the *ycf1* gene its location in the various species was similar to that in *A. thaliana* except for the cases of *Aethionema grandiflorum*, *Aethionema arabicum*, *Breschneidera sinensis*, *Crateva tapia,* and *Salvadora persica* (where the gene is located at the JSB junction), and *M. oleifera* (where it is absent). Likewise, the *psbA* gene was found to be in the LSC region. In contrast, in *Pentadiplandra brazzeana* the *rpl22* gene is in the IRb region. Finally, the position of the *rps19* gene in all related plastomes is close to that in *A. thaliana* except for the cases of *Salvadora persica* (where it can be found in the LSC region), and *Pentadiplandra brazzeana* (where it can be found in IRa and IRb regions).

### Plastome structural variations

**FIGURE 3:**
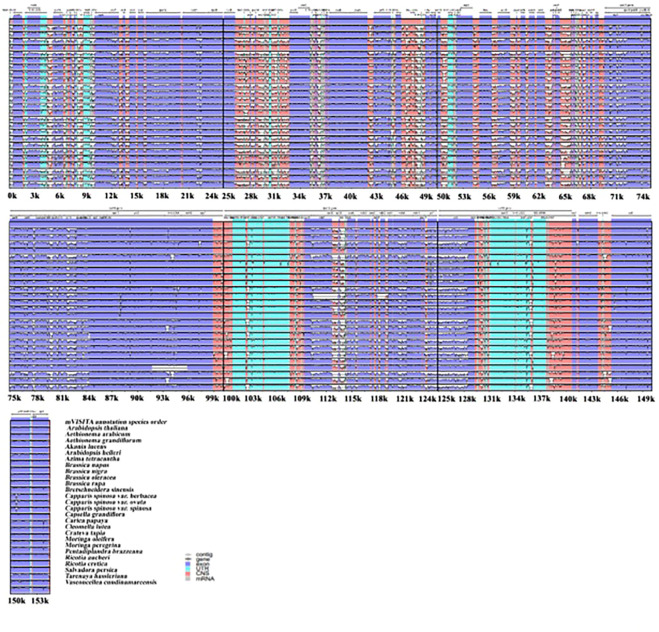
Plastomes genomic variations for the 26 relative speices using mVISITA software. The above gray axis obtained genes’ position and order in plastome structures and LCS, SSC, and IRs regions.

To investigate the plastome features of the 26 related species, complete structural alignment was established using *A. thaliana* as the reference for annotation (Figure 3). These species belong to different families within the Brassicales order. The gene orientations and numbers were recorded, and our findings show that coding spots are highly stable compared with non-coding spots among the species. The inverted repeat regions were observed to present fewer variations than single-copy regions. Moreover, the most significant divergent loci in the coding regions were *matK, aacD, rpoC2, rpl20, rpl32, ccsA, ndhD, rps15*, and *ndhF*. On the other hand, in the non-coding regions the principal divergent loci were *psbK-psbI, psbI-trnS (GCU),trnC(GCA)- trnD(GUG), trnY(GUA)- trnT(GGU), psbC- trnS(UGA), trnL(UAA)- rps4, trnV(UAC)- ndhC, aacD-psaI, rpl12-rpl32, rps15-ycf1, trnA(UGC)* and *rps7*. In phylogenetic studies, genome diversity is considered in terms of nucleotide variations, and DNA coding, and non-coding spots (Figure 3).

### Repeats and codon bias in related species

**FIGURE 4:**
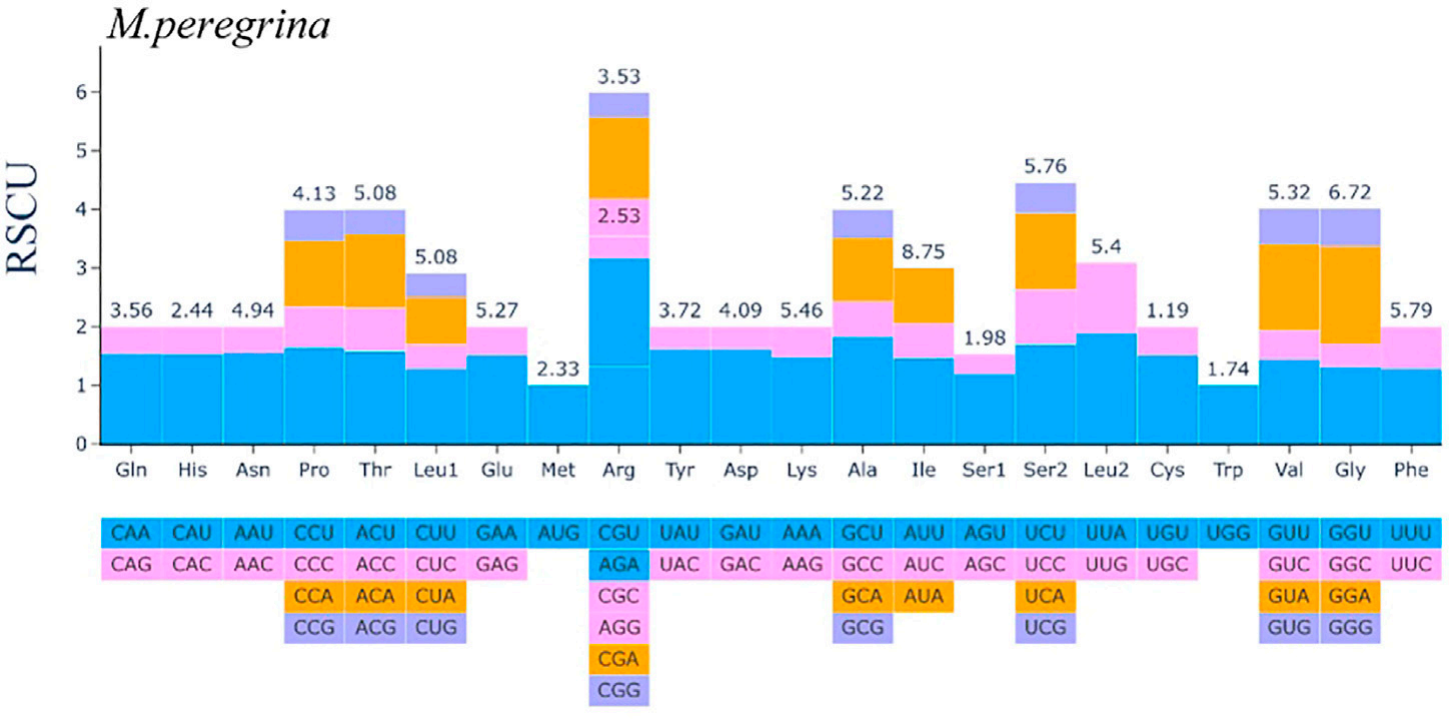
The codon Usage Bias of the plastomes structure of *M. peregrina* in all protein-coding genes.

In this section, we present the results of codon usage analysis; the findings are summarized in (Figure 2; Supplementary Table S1). The results indicate that 20 amino acids can be transported for protein biosynthesis by tRNA in the *M. peregrina* plastome structure. Moreover, all the CDSs consist of 26,665 codons; among them, the codons encoding Isoleucine resulted to be the most used, accounting for 8, 75% of total usage, while the codons encoding Cysteine resulted to be the least used, accounting for 1.19% of total usage in the *M. peregrina* plastome structure. Additionally, as the number of amino acid-encoding codons increases an inevitable increase in the value of RSCU (shortening of relative synonymous codon usage) is also observed, as (Figure 4) shows. Remarkably, the most significant amino acid codons were found in AU(T)G and U(T)GG, encoding methionine and tryptophan, respectively.

**FIGURE 5:**
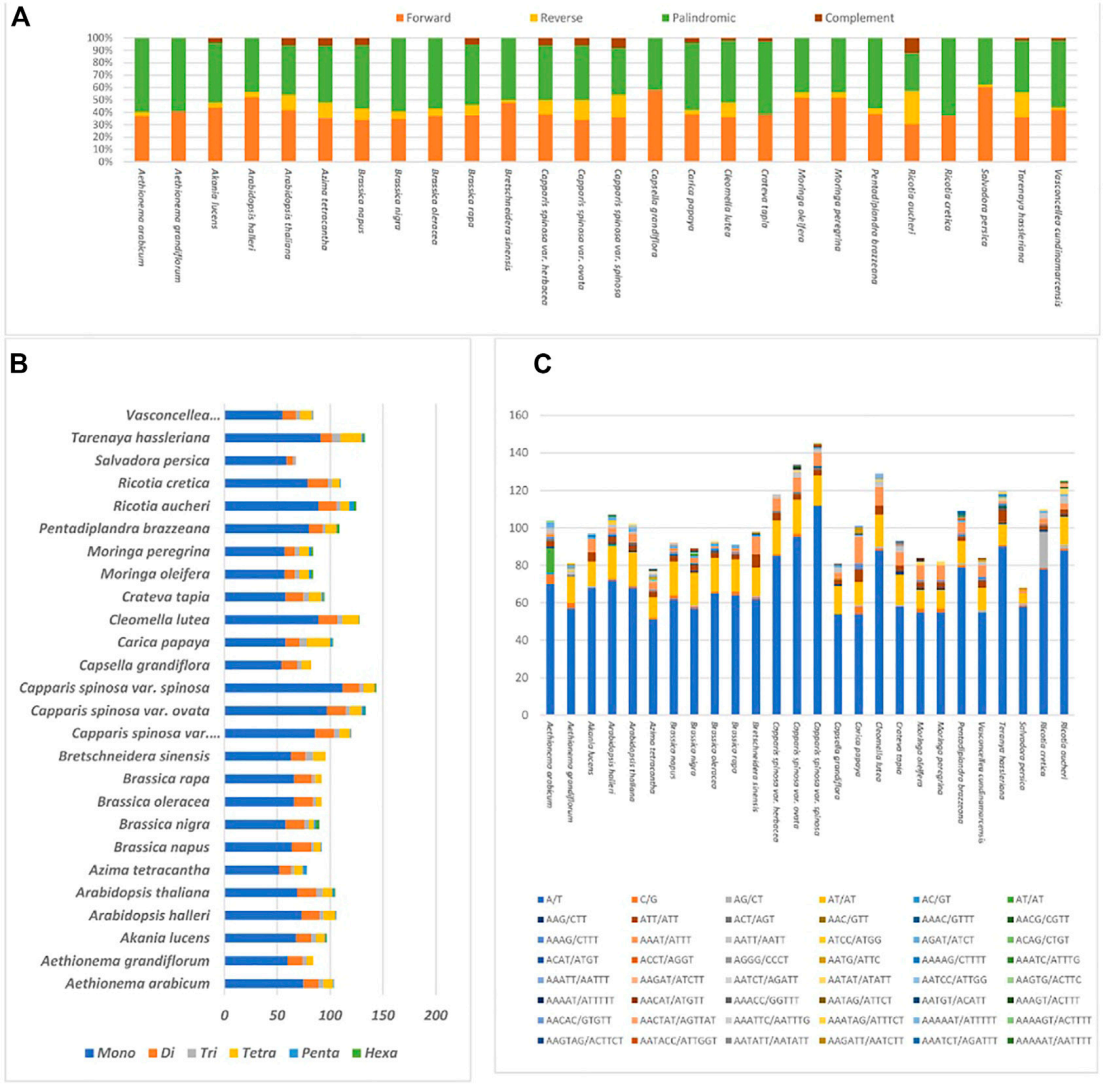
Plastome samples of 26 relative species **(A)**. Total motifs of repeat sequences identified individually **(B)**. Total motifs of various SSrs types estimates in each specie **(C)**. Total types of SSRs motifs in each specie.

Upon future analysis, the repeat structures in the 26 plastome species comprised various long repeats, including palindromic, forward, reverse, and complement repeats (Figure 5. A). The results indicate that *M. oleifera* and *M. Peregrina* contain the same number of repeat types, i.e., 26 forward- 22 palindromic-, and two reverse-type repeats (Figure 5. A). The forward-type repeats were found to be range between 11 repeats (in *S. persica* and *Capsella grandiflora*) and 29 repeats (in *Brassica nigra* and *A. grandiflorum*), while the palindromic-type repeats were found to be range from 13 repeats (in *R. aucheri*) to 27 repeats (in Caricaceae species). The highest number of reverse-type repeats was observed in *R. aucheri*, and the most abundant complement-type repeats were reported in *C.s. var spinosa* (Figure 5A).

The present study investigated the appearance and types of s sequence of *M. peregrina* and 25 related species (Figures 5B, C). The SSR types in the 26 specimens of the plastome sequences tested encompass several types of SSR repeats, including mono, di, tri, tetra, penta, and hexa nucleotides. On average, the number of mononucleotide motifs found was between 112 (*Capparis spinosa var spinosa*) and 52 (*Azima tetracantha*). Dinucleotide was found to range from 6 (in *R. cretica) to 19* (*S. persica)*. The most numerous repeat types of tri and tetranucleotides recorded were 8 (in *T. hassleriana*) and 22 (in *Carica papaya*). The most numerous penta- and hexanucleotides were found in *R. aucheri*. Meanwhile, A/T mononucleotide repeats were found to be the most aboundant across all the plastomes of the related species (Figure 5C). The results indicate that the SSRs among these plastome sequences mainly combine poly-A and poly-T repeats. Hence, they comprise most of the AT multitude in the plastomes of the 26 species.

### Selective pressure analysis

**FIGURE 6:**
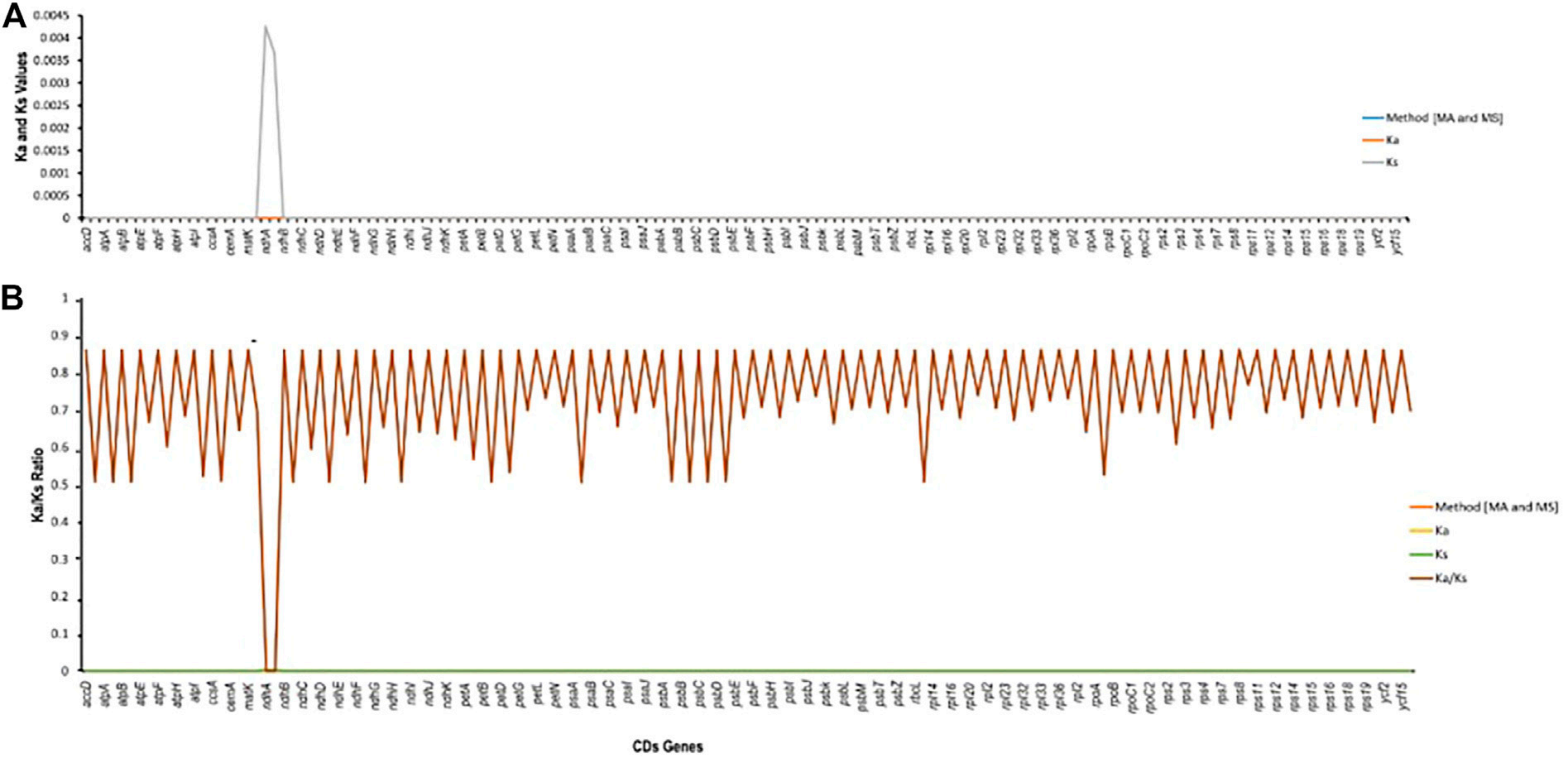
Estimation of CDS in *Moringa oleifera* and *Moribga peregrina*. **(A)** Ka/Ls values. **(B)** Ka/Ks ratio.

The rates of synonymous (Ka) and non-synonymous (Ks) substitution were calculated using a total of 74 regions (protein-coding genes) extracted from the plastomes of *M. peregrina* and *M. oleifera*. In all the extracted regions, the Ks value of *ndhA* (0.00426136) was the highest, and the Ka value of *accD* was slightly the highest among all (0.000000325799). Ka/Ks was also evaluated to determine the effectiveness of selective pressure imposed on specific genes. The Ka/Ks value of shared protein-coding genes suggests that there has been evolutionary pressure to conserve the ancestral state (adverse selection) (see Figure 6); (Ka/Ks = 1, neutral selection; Ka/Ks < 1, purifying selection; and Ka/Ks > 1, positive selection) (IPNI, 2022). Purifying selection is standard in many protein-coding regions. These results indicate that all the genes had a Ka/Ks ratio less than one (Figure 6). Finally, our results suggest that the protein-coding genes in plastomes of different plant species were exposed to diverse selection pressures.

### Phylogenetic analysis

**FIGURE 7:**
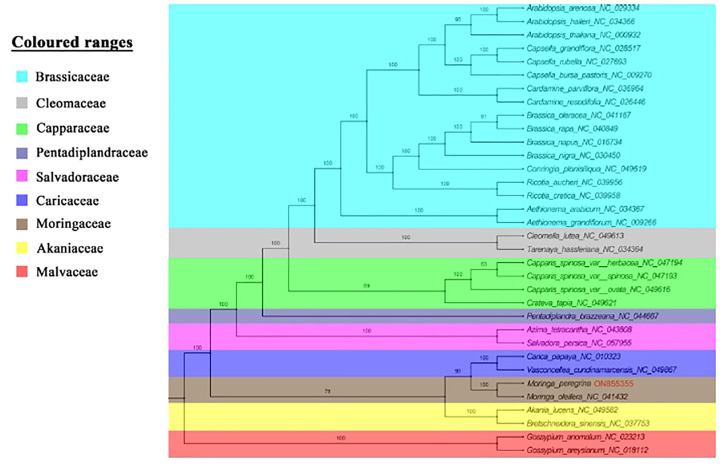
Phylogenetic topology for 34 complete plastome of Brassicales order species and two outgroups depend on 54 protein-coding genes among all plastome structures, used maximum likelihood analysis with bootstrap estimated branches.

The phylogenetics of *M. peregrina* was re-established using 54 shared protein-coding genes from eight family representatives in the order of Brassicales to determine the structure position of *M. peregrina* (Figure 7). The results indicate that all the families in the Brassicales order have been compressed in the sister clade of our phylogenetic tree (Akaniaceae, Caricaecea, Salvadoraceae, Pentadiplandraceae, Capparaceae, Cleomaceae, Brassicaceae, and Moringaceae), except for the Malvaceae family, which belongs to the Malvales order. Additionally, *M. peregrina* forms a sisterhood with *M. oleifera*, as determined using high bootstrap support with the Bayesian method. Moreover, the current study suggests that the Moringaceae family is close to the Caricaceae and Akaniacaeae families.

### Divergence time estimation

**FIGURE 8:**
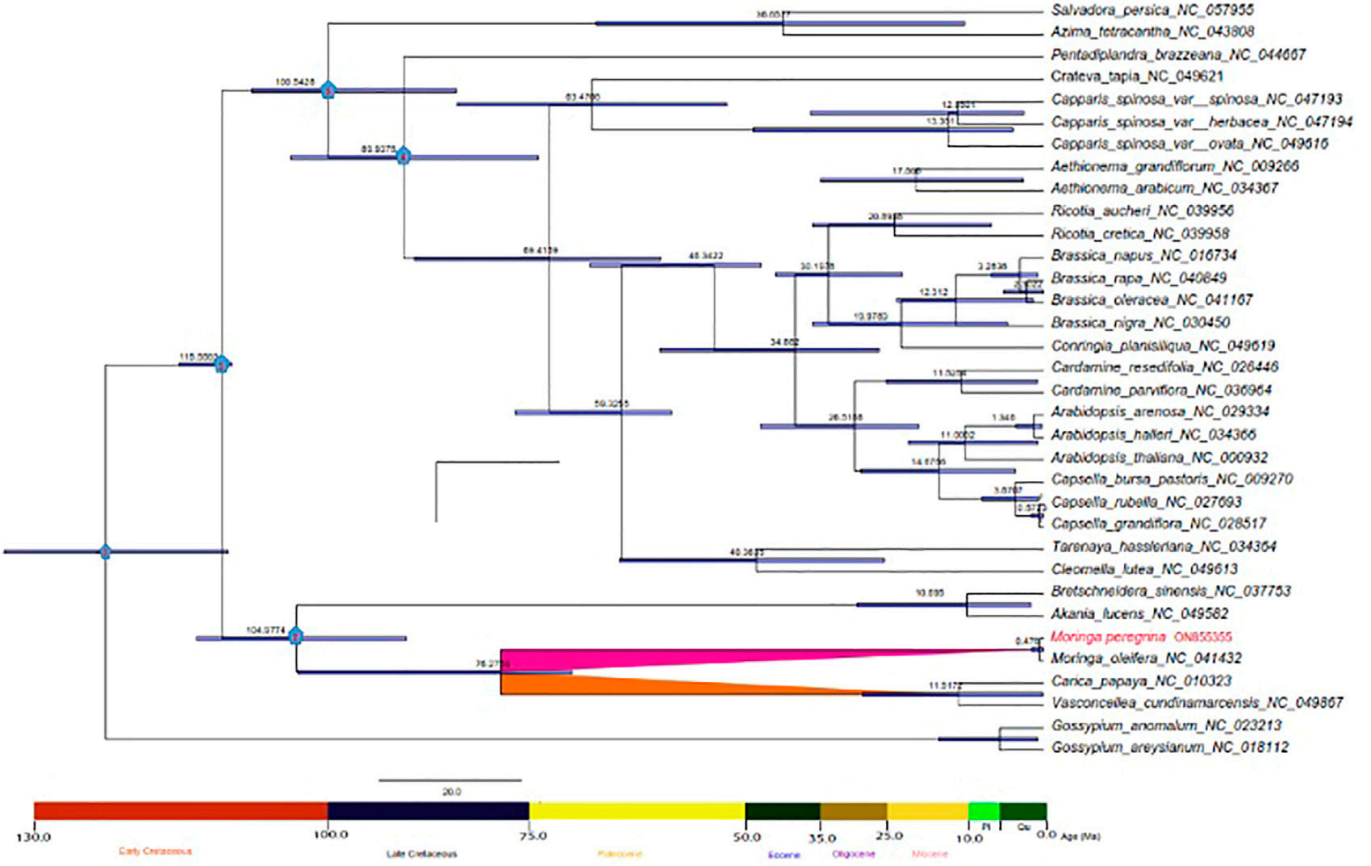
Phylogenetic chronogram showing the evolutionary dating time of order Brassicales using 34 taxa. The tree was estimated using Bayesian analysis of 54 protein-coding genes in the MCMC tree. The number in the circle in red relates to our two nods of interest.

Evolutionary divergence dating time was estimated using 34 taxa of the Brassicales order to calculate the estimated age of the wild type of *M. peregrina* compared with *M. oleifera* and the related taxa. Overall, the root age estimate of Brassicales (node 0) was approximated at 126 million years ago (Ma) (95% HPD; 126.6–116 Ma). The divergence time of Brassicales (node 1) was approximated 115 million years ago (Ma) (95% HPD: 121–114 Ma), which relates to the early Cretaceous junction. The divergence time of the Moringaceae, Caricaceae, and Akaniaceae families (node 2) was approximated at 104 Ma (95% HPD 104–94 Ma) corresponding to the early Cretaceous junction. The Salvadoraceae species divergence time was approximated at 100 Ma (95% HPD: 100 Ma) (node 3), which closely relates to the early Cretaceous junction. In contrast, the divergence time of Core Brassicales (node 4) was approximated at 90 Ma (95% HPD: 90-80 Ma), corresponding to the Late Cretaceous junction. However, the Moringaceae and Caricaceae species diverged 76 Ma (95% HPD: 75-65 Ma), which relates to the Paleocene junction. Finally, *Moringa peregrina* and *Moringa oleifera* diverged 0.476 Ma, indicating recent diversification (Figure 8).

## Discussion

### *Moringa peregrina* plastome structure and comparative variations

In recent years, attention has been paid to the advent of high-throughput sequencing. These genomic tools are crucial for comparative genomics and genome-wide association studies (GWASs). This technology offers opportunities to determine the phylogenetic relationships among closely related species. Recent surveys show that *M. peregrina* has come to be under threat, or even on the border of extinction, primarily due to global climate change and desertification caused by extreme drought (Abdel Raouf et al., 2012). Hence, crucial conservation and restoration actions must be taken to conserve the threatened species *M. peregrina*. To facilitate such purposes, we constructed the first complete plastome structure of wild Egyptian *M. peregrina* to provide a fundamental genetic resource that can be useful to future research on comparative population genomics. Our results reveal high conservation of the cpDNA genome of *M. peregrina* in terms of architecture and linear sequence order. Our results show that the exact length of the *M. peregrina* plastome is similar to the one previously reported in *M. oleifera* (Lin et al., 2019). The total length of the plastomes of related species is ranges from 153,415 bp to 160,600 bp, and the IR length ranges from 25,804 bp to 31,477 bp. The shortest plastome length was recorded in *A. tetracantha*, and the most considerable length was recorded in *M. oleifera* and *M. peregrina*. Moreover, a significant IR length was recorded in *C. Salvadora* var. *herbacea*, while the lowest IR length was recorded in *T. hassleriana*. The LSC length extends from 83,030 bp (in *B. napus)* to 88,749 bp (in *C. papaya)*. Moreover, the SSC length was found to be between 17,488 bp (in *A. tetracantha)* and 18,883 bp (in *M. oleifera* and *M. peregrina).* IR regions are divided by LSC and SSC regions. Our results indicate that greater sequence divergence was observed in LSC and SSC, while fewer sequence differences were found in the two IR regions.

### IR junction variations among related species

In most angiosperm plastomes, the stabilizing sequences mainly lie in the conservative IR region. The contraction and expansion of the IR region can lead to the creation of pseudogenes (Ni et al., 2016a). Our findings show that the *ycf1* gene is located in the JSB region. In contrast, the *rpl22* gene is in the IRb region at the LSC/IRb border, while *rps19* was detected at the LSC/IRa border in all related plastomes. In addition, *rps19* was found to be located in a position close to that in *A. thaliana* but not in *Salvadora persica* (where it is located in the LSC region) or *Pentadiplandra brazzeana* (where it is located in the IRa and IRb regions), possibly due to incomplete duplication and its incapacity to encode proteins. Therefore, the contraction and expansion of the two IR regions represent important evolutionary events responsible for the size differences of the cpDNA genome (Maréchal and Brisson, 2010). This trend is supported by previous results of genome re-sequencing, which revealed that IR regions can be recognized as the key to chloroplast genome evolution, even among narrowly related genera of the same family (Xiong et al., 2009). The results of our comparative analysis of protein-coding gene sequences reveal a wide range of gene numbers. The number of protein-coding genes ranged from 73 (*C. s.* var. *herbacea*) to 88 (*M. peregrina*). The number of tRNA genes was found to be between 36 (*M. oleifera* and *M. peregrina*) and 38(*T. hassleriana*). Further, the number of rRNA genes is similar 8) in all related species; *A. thaliana* does not follow that phenomenon with seven genes (Seol et al., 2017).

### Plastome structural variations among species

The plastome sequences analyzed show great conservation of gene order and composition, as evidenced with mVISTA annotation, which we used to compare the complete plastomes of the species’. It is known that the non-coding genes of the chloroplast genome have diverse signatures. They are responsible for cpDNA genome size variations, which offers superior levels of evolutionary frequency for barcoding and phylogenetic studies at the subspecies level (Amar, 2020). According to our study result, twelve non-coding genes show higher variance than coding genes, agreeing with previous results (Perry and Wolfe, 2002). We detected less hyper-shifting in the coding structures than in the non-coding structures. These findings could be used in future DNA barcode studies; this information on the genetic structure could be helpful in terms of providing parameters for phylogenetic relationships and divergence within various species belonging to the Brassicales order (Edger et al., 2018).

### Repeats and codon bias variations

Simple sequence repeats (SSRs) are vital DNA markers because of features, such as high reproducibility, co-dominant inheritance, abundance, multiallelic nature, and comprehensive genome coverage (Brake et al., 2022). It is well known that repeat sequences have widespread structures in non-coding and coding regions of the cpDNA genome, playing a vital role in plastome recombination, which is used as a parameter in phylogenetics, population genetics analyses, and evolutionary studies (Cavalier-Smith, 2002; Ni et al., 2016b). In the present study, we used REPuter software and the MISA application to evaluate the repeat sequences in the whole-plastome sequence of *M. peregrina*, Herein, numerous polymorphic SSR repeat units of between one and six nucleotides were identified in *M. peregrina* with an average length between 30 and 90 bp, corresponding to other previously recorded angiosperm lengths (Li et al., 2017; Greiner et al., 2019). The frequency of genic SSRs found is higher than what previously reported in other Moringaceae (Kaila et al., 2017). These genic SSRs may be potential genetic markers suitable for phylogenetic and population genetic studies in the Brassicales order (Li et al., 2017). All of the above results show that the plastome of wild-type of *M. peregrina* involves some highly diverged hotspot regions, which may be suitable for DNA barcoding, phylogenetics, and molecular evolution studies in the Brassicales order. In the same context, codon usage analysis is essential recognizing the selection pressure on genes and the evolution of the plastome genome structure (Yang et al., 2014). In the current analyses, the usage of the codon encoding Isoleucine was the highest in the *M. peregrina* plastome structure. The results of the RSCU of the codon bias are consistent with research on other angiosperm chloroplast genomes (Asaf et al., 2017; Fan and Ma., 2022). Statistical procedures for performing selective pressure analysis are now applicable to whole-plastome genome data, which aids the identification of alleles that may be vital to our understanding of plant evolution. Overall, the selective pressure analysis results vary considerably across genes. However, the accelerated rate of amino acid substitutions in a particular gene during a particular evolutionary period is evidence that the gene plays an essential role in adaptive evolution in a given species.

### Selective pressure analysis in both *moringa* species

The synonymous (Ks) and non-synonymous (Ka) substitution rates, as well as the corresponding ratio (Ka/Ks): Also known as (dN/dS), are widely used to calculate nucleotide evolution rates and natural selection pressure (Yang, 2005). Previous research has found that the Ka/Ks ratio is typically less than one (Yang et al., 2019), owing to synonymous nucleotide substitution rates that are higher than non-synonymous substitution rates. In this study, for all genes the ratio was below one, indicating purifying selection and positive selection (Wu et al., 2020; Zhao et al., 2020). Our results show that two genes named *ndhA* and *accD,* have the highest Ka/Ks variability due to the highest ratio of transitions to transversions. These genes may play an essential role in shaping plastome evolution, as the substitution of transition to transversion commonly affects amino acid mutations in the chloroplast genome (Amar, 2020). The functions of the above genes’ are mainly related to subunits of NADH dehydrogenase and the Acetyl CoA-carboxylase subunit and cytochrome synthesis. They may be under large positive selection due to specific environmental conditions (Huang et al., 2022). Indeed, both genes can be used as candidate barcodes for different species and to perform phylogenetic framework and systematic revision in future studies.

### Phylogenetic analysis

Earlier studies of Brassicales families have shown an adequate comprehension of phylogenetic relationships among families as established with molecular, morphology, and taxonomic analyses; however, the arrangement of the Moringaceae has not been fully determined (Karol et al., 1999; Olson and Carlquist, 2001; Lin et al., 2019). Compared with fragment DNA markers, plastomes have been shown to provide sufficient phylogenetic signals, which are important for determining deep relationships of plant lineage (Gitzendanner et al., 2018). A recent phylogenetic analysis used a limited number of species and gene regions as Moringaceae placeholders (Lin et al., 2019; Yang et al., 2019). In general, the topological structure of the five phylogenetic trees was similar to the tested accessions but with slight resolution. Our plastome phylogenetic studies determined eight families representing the Brassicales order with high support. As seen in our chloroplast phylogenomic tree the Cleomaceae and the Capparaceae form a particular monophyletic clade. El Zayat et al. (2020) clarified the complex interactions among them, supporting the undisputed viewpoint that the Capparaceae are very closely associated with the Cleomaceae in a sister clade and seem to be distinguished. These results might be applied in systematic and evolutionary biology studies in the Capparis and Cleome. We observed that the Caricaceae and the Moringaceae are clustered with the Akaniaceae, suggesting that they could have a common ancestor, which is in agreement with previous studies (Olson, 2002; Lin et al., 2019; Khan et al., 2021). Our results establish *M. peregrina* and *M. oleifera* as monophyletic clades in the Brassicales order, suggesting that the introgression of the wild types into the chloroplast genome cultivated *Moringa* might have occurred. Indeed, compared with previous analyses on a small quantity of plastome structure fragments, our findings clarify the complex interactions among the narrowly related species of the Brassicales order, providing an effectively determined phylogenetic relationship (Lin et al., 2019; Khan et al., 2021).

### Divergence time estimation

In this study, we selected two reliable fossils representing the early dating time of the Brassicale’s order, which was well-documented in previous studies on angiosperm tree age. Our crown age estimation of Brassicales infers values similar to those reported in previous studies ranging from 121 to 114 Ma (Beilstein et al., 2010; Edger et al., 2015; Magallón et al., 2015; Cardinal-McTeague et al., 2016). Previous lines of evidence place the Caricaceae and the Akaniaceae with the Moringaceae and within the Brassicales order. This concept has gained much acceptance and support the evidence recommended by Beilstein et al. (2010); a hypothesis is that these placements have fueled speculations that genome-doubling events are linked with diversification in the Brassicaceae. The time divergence period of the Moringaceae was estimated to be around the Oligocene-Paleocene periods, which are linked to the development of Brassicales phylogeny in angiosperms (Kagale et al., 2014; Rockinger et al., 2016). Our results suggest that both genome structure and developmental processes have evolved slower than appreciated. The evolutionary history of *Moringa peregrina* and *Moringa oleifera* and their neighborhood here described could allow a more precise application of our understanding of this model organism to other flowering plants in the Brassicales order to be achieved. Finally, the estimated age of *Moringa peregrina* and *Moringa oleifera* (0.476 Ma) is older than previously estimated (Cardinal-McTeague et al., 2016), possibly resulting from our use of multiple analyses or dense taxon sampling within the Brassicales order. Furthermore, using additional species from the Moringaceae family and Brassicales order families is essential to better calculate age estimation, evolution history, and phylogenetic framework. Based on the above considerations, our findings could be useful to future analyses of the whole-plastome sequences and shared protein-coding genes in all referred species. The present study provides the first phylogenomic framework containing the Egyptian wild-type *M. peregrina* plastome.

## Conclusion

The complete plastome structure of the Egyptian wild type of *M. peregrina* is presented here. The study of the *M. peregrina* plastome, together with those of 25 related species belonging to eight families in the Brassicales order, revealed variations in their plastome sequences and composition. The comparative genomic variations were estimated producing data about the Brassicales order by studying plastome diversity-related structures and providing knowledge to understand plastome structural polymorphisms. Useful genomic markers such as SSR repeat sequences and codon usage bias may be helpful in DNA barcoding and evolutionary studies in the Brassicales order, which has yet to be determined. The phylogenetic tree derived from 54 shared protein-coding genes (CDSs) within 34 species reveals that *M. peregrina* and *M. oleifera* form a sisterhood relationship this could be a helpful phylogenetic framework for future studies. The time evolutionary tree suggests that the two *Moringa* species diversified at 0.467 Ma. The collected comparative genetic information, phylogeny, and time diversity estimation here reported provide novel insights into the plastome evolution of *M. peregrina* within the Brassicales order.

## Data Availability

The datasets presented in this study can be found in online repositories. The names of the repository/repositories and accession number(s) can be found below: https://www.ncbi.nlm.nih.gov/, ON855355.

## References

[B1] Abd Rani N. Z. Husain K. Kumolosasi E. (2018). Moringa genus: A review of phytochemistry and pharmacology. Front. Pharmacol. 9, 108. 10.3389/fphar.2018.00108 29503616 PMC5820334

[B2] Abdel-Hameed U. (2015). Molecular phylogenetics of Moringaceae martinov with emphasis on ethnomedicinal plant Moringa oleifera Lam. grown in Egypt. Retrieved from.

[B3] Abdel-Raouf N. Al-Homaidan A. A. Ibraheem I. (2012). Microalgae and wastewater treatment. Saudi J. Biol. Sci. 19 (3), 257–275. 10.1016/j.sjbs.2012.04.005 24936135 PMC4052567

[B4] Al‐Kahtani H. A. (1995). Some antinutritional factors in *Moringa peregrina* (Al‐Yassar or Al‐Ban) and soybean products. J. food Sci. 60 (2), 395–398. 10.1111/j.1365-2621.1995.tb05680.x

[B5] Amaglo N. K. Bennett R. N. Curto R. B. L. Rosa E. A. Turco V. L. Giuffrida A. (2010). Profiling selected phytochemicals and nutrients in different tissues of the multipurpose tree *Moringa oleifera* L., grown in Ghana. Food Chem. 122 (4), 1047–1054. 10.1016/j.foodchem.2010.03.073

[B6] Amar M. H. (2020). *ycf*1-ndhF genes, the most promising plastid genomic barcode, sheds light on phylogeny at low taxonomic levels in *Prunus persica* . J. Genet. Eng. Biotechnol. 18 (1), 42–10. 10.1186/s43141-020-00057-3 32797323 PMC7427673

[B7] Amiryousefi A. Hyvönen J. Poczai P. (2018). IRscope: An online program to visualize the junction sites of chloroplast genomes. Bioinformatics 34 (17), 3030–3031. 10.1093/bioinformatics/bty220 29659705

[B8] Asaf S. Khan A. L. Khan A. R. Waqas M. Kang S.-M. Khan M. A. (2016). Complete chloroplast genome of *Nicotiana otophora* and its comparison with related species. Front. Plant Sci. 7, 843. 10.3389/fpls.2016.00843 27379132 PMC4906380

[B9] Asaf W. M. Khan A. L. Khan M. A. Kang S. M. Imran Q. M. Shahzad R. (2017). The complete chloroplast genome of wild rice (Oryza minuta) and its comparison to related species. Front. plant Sci. 7 (8), 304. 10.3389/fpls.2017.00304 PMC5339285 28326093

[B10] Barbosa M. S. Freire C. C. Almeida L. C. Freitas L. S. Souza R. L. Pereira E. B. (2019). Optimization of the enzymatic hydrolysis of *Moringa oleifera* Lam oil using molecular docking analysis for fatty acid specificity. Biotechnol. Appl. Biochem. 66 (5), 823–832. 10.1002/bab.1793 31206795

[B11] Beier S. Thiel T. Münch T. Scholz U. Mascher M. (2017). MISA-Web: A web server for microsatellite prediction. Bioinformatics 33, 2583–2585. 10.1093/bioinformatics/btx198 28398459 PMC5870701

[B12] Beilstein M. A. Nagalingum N. S. Clements M. D. Manchester S. R. a. M., S. Mathews S. (2010). Dated molecular phylogenies indicate a Miocene origin for *Arabidopsis thaliana* . Proc. Natl. Acad. Sci. 107 (43), 18724–18728. 10.1073/pnas.0909766107 20921408 PMC2973009

[B14] Boopathi N. M. Abubakar B. Y. (2021). “Botanical descriptions of moringa spp,” in The Moringa genome (Springer), 11–20.

[B15] Brake M. Al-Qadumii L. Hamasha H. Migdadi H. Awad A. Haddad N. (2022). Development of SSR markers linked to stress responsive genes along tomato chromosome 3 (Solanum lycopersicum L.). BioTech 11 (3), 34. 10.3390/biotech11030034 35997342 PMC9397033

[B16] Brenner G. J. (1996). “Evidence for the earliest stage of angiosperm pollen evolution: A paleoequatorial section from Israel,” in Flowering plant origin, evolution & phylogeny (Boston, MA: Springer), 91–115.

[B17] Brudno M. Do C. B. Cooper G. M. Kim M. F. Davydov E. Green E. D. (2003). LAGAN and multi-LAGAN: Efficient tools for large-scale multiple alignment of genomic DNA. Genome Res. 13 (4), 721–731. 10.1101/gr.926603 12654723 PMC430158

[B18] Cardinal-McTeague W. M. Sytsma K. J. Hall J. C. (2016). Biogeography and diversification of brassicales: A 103 million year tale. Mol. Phylogenetics Evol. 99, 204–224. 10.1016/j.ympev.2016.02.021 26993763

[B19] Cavalier-Smith T. (2002). Chloroplast evolution: Secondary symbiogenesis and multiple losses. Curr. Biol. 12, R62–R64. 10.1016/s0960-9822(01)00675-3 11818081

[B20] Doyle J. (1991). DNA protocols for plants. Molecular techniques in taxonomy. Springer.

[B21] Drummond A. J. Suchard M. A. Xie D. Rambaut A. (2012). Bayesian phylogenetics with BEAUti and the BEAST 1.7. Mol. Biol. Evol. 29 (8), 1969–1973. 10.1093/molbev/mss075 22367748 PMC3408070

[B23] Ebert A. Olson M. Bates R. Palada M. (2019). “Genetic resources, diversity and crop improvement,” in The miracle tree: Moringa oleifera.

[B24] Edger P. P. Heidel-Fischer H. M. Bekaert M. Rota J. Glöckner G. Platts A. E. (2015). The butterfly plant arms race escalated by gene and genome duplications. Proc. Natl. Acad. Sci. 112 (27), 8362–8366. 10.1073/pnas.1503926112 26100883 PMC4500235

[B25] Edger P. P. Hall J. C. Harkess A. Tang M. Coombs J. Mohammadin S. (2018). Brassicales phylogeny inferred from 72 plastid genes: A reanalysis of the phylogenetic localization of two paleopolyploid events and origin of novel chemical defenses. Am. J. Bot. 105 (3), 463–469. 10.1002/ajb2.1040 29574686

[B26] El Sohaimy S. A. Hamad G. M. Mohamed S. E. Amar M. H. Al-Hindi R. R. (2015). Biochemical and functional properties of *Moringa oleifera* leaves and their potential as a functional food. Glob. Adv. Res. J. Agric. Sci. 4 (4), 188–199.

[B27] El Zayat M. A. S. Ali M. E. S. Amar M. H. (2020). A systematic revision of *Capparaceae* and *Cleomaceae* in Egypt: An evaluation of the generic delimitations of Capparis and Cleome using ecological and genetic diversity. J. Genet. Eng. Biotechnol. 18, 58–15. 10.1186/s43141-020-00069-z 33025275 PMC7538494

[B28] Elsayed E. Farooq M. Sharaf-Eldin M. El-Enshasy H. Wadaan M. (2020). Evaluation of developmental toxicity and anti-angiogenic potential of essential oils from *Moringa oleifera* and *Moringa peregrina* seeds in zebrafish (*Danio rerio*) model. South Afr. J. Bot. 129, 229–237. 10.1016/j.sajb.2019.07.022

[B29] Fahey J. W. (2005). *Moringa oleifera*: A review of the medical evidence for its nutritional, therapeutic, and prophylactic properties. Part 1. Trees life J. 1 (5), 1–15.

[B30] Fan Z. F. Ma C. L. (2022). Comparative chloroplast genome and phylogenetic analyses of Chinese Polyspora. Sci. Rep. 12 (15984), 1–15. 10.1038/s41598-022-16290-4 36163343 PMC9512918

[B31] Frazer K. A. Pachter L. Poliakov A. Rubin E. M. Dubchak I. (2004). Vista: Computational tools for comparative. Genomics. Nucleic Acids Res. 32, W273–W279. 10.1093/nar/gkh458 15215394 PMC441596

[B32] Gitzendanner M. A. Soltis P. S. Yi T. S. Li D. Z. Soltis D. E. (2018). Plastome phylogenetics: 30 years of inferences into plant evolution. Adv. botanical Res. 85, 293–313. 10.1016/bs.abr.2017.11.016

[B33] Greiner S. Lehwark P. Bock R. (2019). OrganellarGenomeDRAW (OGDRAW) version 1.3.1: Expanded toolkit for the graphical visualization of organellar genomes. Nucleic Acids Res. 47, W59–W64. 10.1093/nar/gkz238 30949694 PMC6602502

[B34] Group A. P. (2009). An update of the angiosperm phylogeny group classification for the orders and families of flowering plants: Apg III. Botanical J. Linn. Soc. 161 (2), 105–121. 10.1111/j.1095-8339.2009.00996.x

[B35] Group A. P. Chase M. W. Christenhusz M. J. Fay M. F. Byng J. Judd W. (2016). An update of the angiosperm phylogeny group classification for the orders and families of flowering plants: Apg IV. Botanical J. Linn. Soc. 181 (1), 1–20. 10.1111/boj.12385

[B36] Guindon S. Dufayard J. F. Lefort V. Anisimova M. Hordijk W. Gascuel O. (2010). New algorithms and methods to estimate maximum-likelihood phylogenies: Assessing the performance of PhyML 3.0. Syst. Biol. 59 (3), 307–321. 10.1093/sysbio/syq010 20525638

[B37] Ho S. Y. Phillips M. J. (2009). Accounting for calibration uncertainty in phylogenetic estimation of evolutionary divergence times. Syst. Biol. 58 (3), 367–380. 10.1093/sysbio/syp035 20525591

[B38] Huang X. Coulibaly D. Tan W. Ni Z. Shi T. Li H. (2022). The analysis of genetic structure and characteristics of the chloroplast genome in different Japanese apricot germplasm populations. BMC Plant Biol. 22 (354), 1–13. 10.1186/s12870-022-03731-5 35864441 PMC9306182

[B39] Jaja-Chimedza A. Graf B. L. Simmler C. Kim Y. Kuhn P. Pauli G. F. (2017). Biochemical characterization and anti-inflammatory properties of an isothiocyanate-enriched moringa (Moringa oleifera) seed extract. PloS one 12 (8), e0182658. 10.1371/journal.pone.0182658 28792522 PMC5549737

[B40] Jin J.-J. Yu W.-B. Yang J.-B. Song Y. Depamphilis C. W. Yi T.-S. (2020). GetOrganelle: A fast and versatile toolkit for accurate de novo assembly of organelle genomes. Genome Biol. 21 (241), 1–31. 10.1186/s13059-020-02154-5 PMC7488116 32912315

[B41] Kagale S. Robinson S. J. Nixon J. Xiao R. Huebert T. Condie J. (2014). Polyploid evolution of the Brassicaceae during the cenozoic era. Plant Cell. 26 (7), 2777–2791. 10.1105/tpc.114.126391 25035408 PMC4145113

[B42] Kaila T. Chaduvla P. K. Rawal H. C. Saxena S. Tyagi A. Mithra S. (2017). Chloroplast genome sequence of cluster bean (*Cyamopsis tetragonoloba* L.): Genome structure and comparative analysis. Genes. 8, 212. 10.3390/genes8090212 28925932 PMC5615346

[B43] Kalyaanamoorthy S. Minh B. Q. Wong T. K. Von Haeseler A. Jermiin L. S. (2017). ModelFinder: Fast model selection for accurate phylogenetic estimates. Nat. methods. 14 (6), 587–589. 10.1038/nmeth.4285 28481363 PMC5453245

[B44] Karol K. G. Rodman J. E. Conti E. Sytsma K. J. (1999). Nucleotide sequence of *rbcL* and phylogenetic relationships of Setchellanthus caeruleus (Setchellanthaceae). Taxon 48 (2), 303–315. 10.2307/1224435

[B45] Katoh K. Standley D. (2013). MAFFT multiple sequence alignment software version 7: Improvements in performance and usability. Mol. Biol. Evol. 30 (4), 772–780. 10.1093/molbev/mst010 23329690 PMC3603318

[B46] Kearse M. Moir R. Wilson A. Stones-Havas S. Cheung M. Sturrock S. (2012). Geneious basic: An integrated and extendable desktop software platform for the organization and analysis of sequence data. Bioinformatics 28, 1647–1649. 10.1093/bioinformatics/bts199 22543367 PMC3371832

[B47] Khan A. Asaf S. Khan A. L. Al-Harrasi A. Al-Sudairy O. Abdulkareem N. M. (2019). First complete chloroplast genomics and comparative phylogenetic analysis of *Commiphora gileadensis* and *C. foliacea*: Myrrh-producing trees. PloS One 14, e0208511. 10.1371/journal.pone.0208511 30629590 PMC6328178

[B48] Khan A. L. Asaf S. Al-Rawahi A. Al-Harrasi A. (2021). Decoding first complete chloroplast genome of toothbrush tree (*Salvadora persica* L.): Insight into genome evolution, sequence divergence and phylogenetic relationship within brassicales. BMC Genomics 22, 312–316. 10.1186/s12864-021-07626-x 33926374 PMC8086069

[B49] Kurtz S. Choudhuri J. V. Ohlebusch E. Schleiermacher C. Stoye J. Giegerich R. (2001). REPuter: The manifold applications of repeat analysis on a genomic scale. Nucleic acids Res. 29 (22), 4633–4642. 10.1093/nar/29.22.4633 11713313 PMC92531

[B50] Leite P. M. Castilho R. O. (2017). Chemosystematics of *brassicales* . Biochem. Syst. Ecol. 71, 205–211. 10.1016/j.bse.2017.02.011

[B51] Li J. Zhang Z. Vang S. Yu J. Wong G. K. S. Wang J. (2009). Correlation between Ka/Ks and Ks is related to substitution model and evolutionary lineage. J. Mol. Evol. 68 (4), 414–423. 10.1007/s00239-009-9222-9 19308632

[B52] Li Y. Xu W. Zou W. iang D. Liu X. (2017). Complete chloroplast genome sequences of two endangered Phoebe (*Lauraceae*) species. Bot. Stud. 58, 37–10. 10.1186/s40529-017-0192-8 28905330 PMC5597560

[B53] Lin W. Dai S. Chen Y. Zhou Y. Liu X. (2019). The complete chloroplast genome sequence of *Moringa oleifera* Lam. (Moringaceae). Mitochondrial DNA Part B 4, 4094–4095. 10.1080/23802359.2019.1627922 33366334 PMC7687391

[B54] Liu C. Shi L. Zhu Y. Chen H. Lin X. Guan X. (2012). CpGAVAS, an integrated web server for the annotation, visualization, analysis, and GenBank submission of completely sequenced chloroplast genome sequences. BMC Genomics 13 (1), 715–717. 10.1186/1471-2164-13-715 23256920 PMC3543216

[B55] Liu J. Cai H.-H. Li H.-Q. Liu Z.-Y. Zheng C. Shi C. (2019). The chloroplast genome of *Moringa oleifera* (Moringaceae). Mitochondrial DNA Part B 4 (1), 646–647. 10.1080/23802359.2018.1545550

[B56] Magallón S. Gómez‐Acevedo S. Sánchez‐Reyes L. L. Hernández‐Hernández T. (2015). A metacalibrated time‐tree documents the early rise of flowering plant phylogenetic diversity. New Phytol. 207 (2), 437–453. 10.1111/nph.13264 25615647

[B57] Mansour M. Mohamed M. F. Elhalwagi A. El-Itriby H. A. Shawki H. H. Abdelhamid I. A. (2019). Research article *Moringa peregrina* leaves extracts induce apoptosis and cell cycle arrest of hepatocellular carcinoma. BioMed Res. Int. 2019, 13. 10.1155/2019/2698570 PMC6332967 30713850

[B58] Maréchal A. Brisson N. (2010). Recombination and the maintenance of plant organelle genome stability. New Phytol. 186, 299–317. 10.1111/j.1469-8137.2010.03195.x 20180912

[B59] Menon S. Al Mamari H. K. Al Zaabi H. H. Al Ajmi Z. S. Al Haddabi L. H. Jayachandran V. (2021). Evaluation of the effect of *Moringa peregrina* bark on the crystal habit and size of calcium oxalate monohydrate crystals in different stages of crystallization using experimental and theoretical methods. CrystEngComm 23 (14), 2673–2682. 10.1039/d1ce00080b

[B60] Minh B. Q. Nguyen M. A. von Haeseler A. (2013). Ultrafast approximation for phylogenetic bootstrap. Mol. Biol. Evol. 30, 1188–1195. 10.1093/molbev/mst024 23418397 PMC3670741

[B61] Moerman D. (2013). The Global Flora: Descriptive statistics with a commentary and an ethnobotanical example.

[B62] Ni L. Zhao Z. Dorje G. Ma M. (2016a). The complete chloroplast genome of Ye-Xing-Ba (*Scrophularia dentata; Scrophulariaceae*), an alpine Tibetan herb. PLoS ONE 11, e0158488. 10.1371/journal.pone.0158488 27391235 PMC4938499

[B63] Ni L. Zhao Z. Xu H. Chen S. Dorje G. (2016b). The complete chloroplast genome of *Gentiana straminea* (Gentianaceae), an endemic species to the Sino-Himalayan subregion. Gene 577, 281–288. 10.1016/j.gene.2015.12.005 26680100

[B64] Olson M. Carlquist S. (2001). Stem and root anatomical correlations with life form diversity, ecology, and systematics in Moringa (Moringaceae). Botanical J. Linn. Soc. 135 (4), 315–348. 10.1111/j.1095-8339.2001.tb00786.x

[B65] Olson M. Committee F. o. N. A. E. (1993). “Moringaceae martinov,” in Drumstick tree family (Flora of North America), 167–169.

[B66] Olson M. E. (2002). Combining data from DNA sequences and morphology for a phylogeny of *Moringaceae* (*Brassicales*). Syst. Bot. 27 (1), 55–73. 10.1043/0363-6445-27.1.55

[B88] Padayachee B. Baijnath H. (2012). An overview of the medicinal importance of Moringaceae. J. Med. Plant Res. 6 (48), 5831–5839.

[B67] Perry A. S. Wolfe K. H. (2002). Nucleotide substitution rates in legume chloroplast DNA depend on the presence of the inverted repeat. J. Mol. Evol. 55 (5), 501–508. 10.1007/s00239-002-2333-y 12399924

[B68] Qu X.-J. Moore M. J. Li D.-Z. Yi T.-S. (2019). Pga: A software package for rapid, accurate, and flexible batch annotation of plastomes. Plant Methods 15, 50–12. 10.1186/s13007-019-0435-7 31139240 PMC6528300

[B69] Rizk M. S. Hassan A. El Zayat M. A. S. (2021). Genetic analysis of cytochrome b6 and cytochrome f genes in Egyptian *moringa peregrina*, a threatened wild medicinal plant. Plant Cell. Biotechnol. Mol. Biol. 22, 80–95.

[B70] Rockinger A. Sousa A. Carvalho F. A. Renner S. S. (2016). Chromosome number reduction in the sister clade *of Carica papaya* with concomitant genome size doubling. Am. J. Bot. 103 (6), 1082–1088. 10.3732/ajb.1600134 27234227

[B72] Seol Y.-J. Kim K. Kang S.-H. Perumal S. Lee J. Kim C.-K. (2017). The complete chloroplast genome of two Brassica species, *Brassica nigra* and *B. oleracea* . Mitochondrial DNA Part A 28, 167–168. 10.3109/19401736.2015.1115493 26709541

[B73] Singh B. P. Kumar A. Kaur H. Singh H. Nagpal A. K. (2020). CpGDB: A comprehensive database of chloroplast genomes. Bioinformation 16, 171–175. 10.6026/97320630016171 32405169 PMC7196173

[B74] Sodvadiya M. Patel H. Mishra A. Nair S. (2020). Emerging insights into anticancer chemopreventive activities of nutraceutical *Moringa oleifera*: Molecular mechanisms, signal transduction and *in vivo* efficacy. Curr. Pharmacol. Rep. 6 (2), 38–51. 10.1007/s40495-020-00210-z

[B75] Sun G. Dilcher D. L. Wang H. Chen Z. (2011). A eudicot from the early cretaceous of China. Nature 471 (7340), 625–628. 10.1038/nature09811 21455178

[B76] Tillich M. Lehwark P. Pellizzer T. Ulbricht-Jones E. S. Fischer A. Bock R. (2017). GeSeq–versatile and accurate annotation of organelle genomes. Nucleic Acids Res. 45, W6–W11. 10.1093/nar/gkx391 28486635 PMC5570176

[B77] Verdcourt B. (1986). Flora of tropical east africa-moringaceae (1986). CRC Press.

[B78] Wilkie P. Poulsen A. D. Harris D. Forrest L. L. (2013). The collection and storage of plant material for DNA extraction: The teabag method. Gardens’ Bull. Singap. 65, 231–234.

[B79] Wu Z. Liao R. Yang T. Dong X. Lan D. Qin R. (2020). Analysis of six chloroplast genomes provides insight into the evolution of *Chrysosplenium* (Saxifragaceae). BMC Genomics 21 (625), 1–14. 10.1186/s12864-020-07045-4 PMC7488271 32912155

[B80] Xiong A. S. Peng R. H. Zhuang J. Gao F. Zhu B. Fu X. Y. (2009). Gene duplication, transfer, and evolution in the chloroplast genome. Biotechnol. Adv. 27 (4), 340–347. 10.1016/j.biotechadv.2009.01.012 19472510

[B81] Yang X. Luo X. Cai X. (2014). Analysis of codon usage pattern in *Taenia saginata* based on a transcriptome dataset. Parasit. Vectors 7 (527), 1–11. 10.1186/s13071-014-0527-1 25440955 PMC4268816

[B82] Yang Y. Tian Y. He S.-L. (2019). Characterization of the complete chloroplast genome of *Moringa oleifera* Lam. (Moringaceae), an important edible species in India. Mitochondrial DNA Part B 4, 1913–1915. 10.1080/23802359.2019.1611393

[B83] Yang Z. (2005). The power of phylogenetic comparison in revealing protein function. Proc. Natl. Acad. Sci. 102 (9), 3179–3180. 10.1073/pnas.0500371102 15728394 PMC552944

[B84] Yule G. U. (1925). A mathematical theory of evolution, based on the conclusions of Dr. JC Willis, FR S. Philosophical transactions of the Royal Society of London. Ser. B, Contain. Pap. a Biol. character 213 (402-410), 21–87. 10.1098/rstb.1925.0002

[B85] Zhang F. Y. T. Wang X. Deng X. Zhang X. Hu S. Yu J. (2012). The complete chloroplast and mitochondrial genome sequences of *Boea hygrometrica*: Insights into the evolution of plant organellar genomes. PLoS One 7 (1), e30531. 10.1371/journal.pone.0030531 22291979 PMC3264610

[B86] Zhang D, D. Gao F. J. akovlić I Z. H. Zhang J L. W. Wang G. T. Li W. X. (2020). PhyloSuite: An integrated and scalable desktop platform for streamlined molecular sequence data management and evolutionary phylogenetics studies. Mol. Ecol. Resour. 20 (1), 348–355. 10.1111/1755-0998.13096 31599058

[B87] Zhao D. N. Ren Y. Zhang J. Q. (2020). Conservation and innovation: Plastome evolution during rapid radiation of *rhodiola* on the qinghai-Tibetan plateau. Mol. Phylogenetics Evol. 144, 106713. 10.1016/j.ympev.2019.106713 31863901

