# Supplementary material for: Comparative genomics analysis of endangered wild Egyptian Moringa peregrina (Forssk.) Fiori plastome, with implications for the evolution of Brassicales order
Source: Front Genet. 2023 Mar 13;14:1131644. doi: 10.3389/fgene.2023.1131644 (PMC10040795; doi:10.3389/fgene.2023.1131644)
Supplement: Supplementary file 1 [file Table1.DOCX]

Supplementary Material

# Supplementary Tables

# Table.1 codon usage percentage

| AA | Count | % |
| --- | --- | --- |
| Phe(F) | 1544 | 5.79 |
| Leu2(L2) | 1440 | 5.4 |
| Leu1(L1) | 1354 | 5.08 |
| Ile(I) | 2333 | 8.75 |
| Met(M) | 621 | 2.33 |
| Val(V) | 1418 | 5.32 |
| Ser2(S2) | 1536 | 5.76 |
| Pro(P) | 1101 | 4.13 |
| Thr(T) | 1354 | 5.08 |
| Ala(A) | 1391 | 5.22 |
| Tyr(Y) | 991 | 3.72 |
| His(H) | 651 | 2.44 |
| Gln(Q) | 949 | 3.56 |
| Asn(N) | 1317 | 4.94 |
| Lys(K) | 1456 | 5.46 |
| Asp(D) | 1091 | 4.09 |
| Glu(E) | 1404 | 5.27 |
| Cys(C) | 316 | 1.19 |
| Trp(W) | 464 | 1.74 |
| Arg(R) | 942 | 3.53 |
| Ser1(S1) | 527 | 1.98 |
| Arg(R) | 674 | 2.53 |
| Gly(G) | 1791 | 6.72 |
| codon end in A or T | 18833 | 70.63 |
| codon end in G or T | 14449 | 54.19 |
| Total | 26665 |  |

# Table. 2 CDS protein coding genes used to draw phylogeny tree

| number | CDS names | number | CDS names |
| --- | --- | --- | --- |
| 1 | accD | 28 | psbK |
| 2 | atpA | 29 | psbL |
| 3 | atpB | 30 | psbM |
| 4 | atpE | 31 | psbT |
| 5 | atpF | 32 | rbcL |
| 6 | atpH | 33 | rpl14 |
| 7 | atpI | 34 | rpl20 |
| 8 | ndhB | 35 | rpl23 |
| 9 | ndhC | 36 | rpl2 |
| 10 | petA | 37 | rpl32 |
| 11 | petB | 38 | rpl33 |
| 12 | petD | 39 | rpl36 |
| 13 | petG | 40 | rpoA |
| 14 | psaA | 41 | rpoB |
| 15 | psaB | 42 | rpoC1 |
| 16 | psaC | 43 | rpoC2 |
| 17 | psaI | 44 | rps11 |
| 18 | psaJ | 45 | rps12 |
| 19 | psbA | 46 | rps14 |
| 20 | psbB | 47 | rps15 |
| 21 | psbC | 48 | rps18 |
| 22 | psbD | 49 | rps19 |
| 23 | psbE | 50 | rps2 |
| 24 | psbF | 51 | rps3 |
| 25 | psbH | 52 | rps4 |
| 26 | psbI | 53 | rps8 |
| 27 | psbJ | 54 | ycf2 |
